# Lamb Wave Scattering Analysis for Interface Damage Detection between a Surface-Mounted Block and Elastic Plate

**DOI:** 10.3390/s21030860

**Published:** 2021-01-28

**Authors:** Mikhail V. Golub, Alisa N. Shpak, Inka Mueller, Sergey I. Fomenko, Claus-Peter Fritzen

**Affiliations:** 1 Institute for Mathematics, Mechanics and Informatics, Kuban State University, 350040 Krasnodar, Russia; alisashpak7@gmail.com (A.N.S.); sfom@yandex.ru (S.I.F.); 2 Institute for Mechanics, Mechatronics and Mechanical Engineering, Bochum University of Applied Sciences, D-44801 Bochum, Germany; inka.mueller@hs-bochum.de; 3 Institute for Mechanics and Mechatronics, University of Siegen, D-57068 Siegen, Germany; Claus-Peter.Fritzen@uni-siegen.de

**Keywords:** guided waves, experiment, computational mechanics, resonance, scattering

## Abstract

Since stringers are often applied in engineering constructions to improve thin-walled structures’ strength, methods for damage detection at the joints between the stringer and the thin-walled structure are necessary. A 2D mathematical model was employed to simulate Lamb wave excitation and sensing via rectangular piezoelectric-wafer active transducers mounted on the surface of an elastic plate with rectangular surface-bonded obstacles (stiffeners) with interface defects. The results of a 2D simulation using the finite element method and the semi-analytical hybrid approach were validated experimentally using laser Doppler vibrometry for fully bonded and semi-debonded rectangular obstacles. A numerical analysis of fundamental Lamb wave scattering via rectangular stiffeners in different bonding states is presented. Two kinds of interfacial defects between the stiffener and the plate are considered: the partial degradation of the adhesive at the interface and an open crack. Damage indices calculated using the data obtained from a sensor are analyzed numerically. The choice of an input impulse function applied at the piezoelectric actuator is discussed from the perspective of the development of guided-wave-based structural health monitoring techniques for damage detection.

## 1. Introduction

Metals are still among the most popular construction materials, and metallic plate- or shell-type components are widely employed for buildings, pipelines, bridges, etc. [1,2,3]. After long-term service, defects may appear in metallic engineering structures. One of the main methods for improving the strength of thin-walled structures, which are commonly used in engineering constructions, is to employ stringers in a proper configuration. Surface-mounted inhomogeneities on the surfaces of metallic plates are also used to manufacture periodic structures called elastic metamaterials, which allow the manipulation of guided wave transmission [4]. Therefore, methods for damage detection are necessary for structures with surface-mounted inhomogeneities.

Ultrasonic guided waves (GWs) propagate at relatively long distances in plate-like structures, and therefore, inspections of large areas are possible with the use of GWs. In recent decades, guided-wave-based structural health monitoring (SHM) has been developed with techniques that allow identification and estimation of cracks, delaminations, debondings, etc. [5,6,7,8]. Since GWs are dispersive, multimodal, and attenuating, their use for the purposes of SHM needs insight into physical phenomena and post-processing of the acquired signals, which have complex waveforms.

This is also especially true for the resulting features that are extracted from signals that are sensed by the SHM system. A large variety of feature extraction techniques exist; an overview is given in [9], which divides them into statistics-based and physics-based procedures, as well as time-frequency analyses, time reversal methods, model-based methods, and methods using artificial intelligence. In many cases, the resulting feature is extracted from the time-domain data as a damage indicator [10,11,12,13]. For damage localization in particular, additional information about location is available. To achieve this, several families of approaches exist [10,14,15,16]. In particular, for the case within this paper, pure damage detection using extracted damage indicators as features is already sufficient for an SHM system.

The presence of surface-mounted obstacles or internal inhomogeneities in the inspected engineering structure makes the theoretical description of the behavior of GWs labor-intensive due to the additional structural complexity and multiple scattering. Therefore, the presence of stiffeners, holes, notches, and other inhomogeneities might reduce the diagnostic potential of GW SHM methods. In [17], the authors investigated physical phenomena related to resonances, GW scattering, and conversion at a rectangular stiffener. A significant influence of omega stringer elements on the surface of an isotropic plate on GW propagation was shown in [7]. Moll et al. [7] demonstrated a damage index (DI) increase with the growth of the reference damage size; they also observed mode conversion and stated that their dataset could be used for detailed probability of detection (POD) studies.

Debonding may occur below the stringer itself due to adhesive degradation or an impact. A common problem is that the disconnection of stringers in stiffened composite structures—where the stringers can be affected by disbondings—leads to a skin–stringer separation, which prevents the collaboration of the structural parts [18]. An SHM system for a stiffened composite structure employing Snell’s Law and negligibility of mode conversion was verified in [19]. This SHM system was applied for a disbonding assessment focusing on the analysis of the wave portion scattered by the stringer to detect changes in the reflections. A comprehensive and detailed comparison of SHM systems based on ultrasonic propagating–scattering guided waves and edge detection of strain profiles from distributed fiber optic sensors is presented in this work in terms of stiffener debonding detection on a full-scale composite wing box panel. Ciminello et al. [18] demonstrated that two GW-based SHM methodologies based on piezoelectric patches and a distributed fiber optic sensor provided successful damage detection in a plate with a stiffener and an excellent agreement with classic nondestructive ultrasonic testing. Sherafat et al. [20] considered GW-based SHM for inspection of a skin–stringer panel made of quasi-isotropic plates bonded together with an adhesive film and presented some guidelines related to the selection of the optimal mode and frequency range for debonding inspection based on a scattering analysis. Zhang et al. [21] proposed the frequency energy ratio mapping method to detect a leakage source’s location for stiffened structures using a sensor network and a mapping matrix formed by the frequency-domain energy ratio vectors.

In general, the choices of the particular joining technique and the form of the stringer strongly depend on the specific application [22]. In this study, a typical GW-based SHM system consisting of piezoelectric-wafer active transducers (PWATs) for actuation and sensing on the surface of a flat metal plate with a rectangular bonded/debonded T-joint is considered. The specific kind of rectangular block implemented in the experiment is not often employed in practice compared to omega stringers or T-/L-shaped stringers. However, the main aim of the study is to investigate the applicability of two-dimensional mathematical models and the possibility for damage indices to indicate defects at the interfaces between stringers and elongated structures. To verify the mathematical model and to exclude deviations caused by imperfections in the production of the experimental specimen, a very simple specimen was designed.

Mathematical models are able to provide results that are in good agreement with experimental results; therefore, they are applicable for the analysis of the development of SHM systems. Benchmark comparisons between guided wave simulations of composites and experimental data can be found in [23]. Of course, three-dimensional models provide more accurate predictions of the experiments. Thus, Leckey et al. [24] implemented a three-dimensional elastodynamic finite integration technique to model Lamb wave scattering for flaws in an aluminum plate, and Luchinsky et al. [25] demonstrated good agreement between experimental and numerical voltage signals from PWATs for a honeycomb plate with impact-induced damage. However, the computational costs related to three-dimensional simulations are much higher compared to those of two-dimensional mathematical models, which can be also very efficient for damage detection. For instance, two-dimensional simulations were used to simulate Lamb wave excitation and sensing by rectangular PWATs in an elastic plate with a rectangular elastic obstacle bonded on the surface of the plate [26] and to estimate the properties of guided waves [27,28,29].

In this study, the standard finite element method (FEM) and an extension of the semi-analytical hybrid approach (SAHA) [17,30] were used for two-dimensional mathematical models. The standard FEM was applied for the simulation of the transient problems, whereas the SAHA, which was in a good agreement with the FEM, was used to compute eigenfrequencies and to analyze mode conversion/reflection by obstacles. The SAHA is based on the boundary integral equation method [31] and the spectral element method (SEM) [32]; these two methods meet in the contact area, where the traction vector is unknown. One can see that the SAHA is based on an idea similar to the so-called global–local approach, where a semi-analytical technique is employed to discretize a semi-infinite or elongated area and the FEM discretizes the “local” portion, which contains scatterers of various kinds, including stiffeners [33,34,35].

Two kinds of defects were considered: the partial degradation of the interface simulated by the spring boundary conditions (SBCs) and a one-sided open crack between the obstacle and the waveguide, simulating a debonding. For the first kind, SBCs were used, since the analytic relations derived in [36,37,38] can be used for the estimation of the severity of damage. It should be noted that the detection of the concentration of micro-cracks is among the current SHM problems. For instance, Wang et al. [5] proposed an SHM method for estimating the growth of micro-sized fatigue cracks by employing a breathing-crack model with a plastic zone to reveal the change in fundamental Lamb waves, and they showed experimentally that the increasing trend of ultrasonic nonlinearity fits very well to the FEM analysis results.

For the analysis of the SHM system’s final output, which enables decision-making for stakeholders, two damage indicators based on statistical feature extraction were applied to the data of the two kinds of defects. The feature extraction algorithms were used to analyze the data of the PWATs used as sensors after excitation with two different excitation signal classes over a variety of frequencies from 120 to 600 kHz.

## 2. Experimental Setup

**Figure 1:**
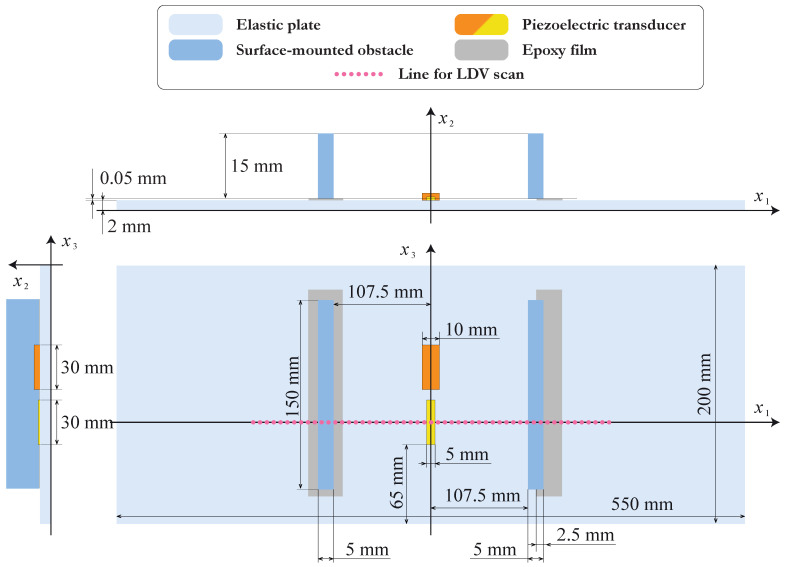
The sketch of the specimen used in the experiment.

In order to investigate the influence of partial debonding between the surface-mounted elastic obstacle and the plate-like structure, the following experiment was conducted. Two obstacles with dimensions 5×15×150 mm were glued at the surface of the 2 mm thick aluminum plate with a thin epoxy film of thickness 0.05 mm. One of the obstacles was fully bonded, while another one was 50% debonded. A sketch of the specimen is presented in Figure 1.

Two piezoelectric transducers were attached at the surface of the plate between the obstacles so that the distances between the centers of both obstacles and the PWATs were equal to 110 mm each. The dimensions of the transducers were 5×30×0.25 mm and 10×30×1 mm. To enable a 2D assumption while modeling, both transducers and obstacles were chosen to be elongated along the $x_{3}$ axis. The measurements were taken from the back side of the plate $x_{2}=0$ along the measurement lines going through the transducers’ centers.

**Figure 2:**
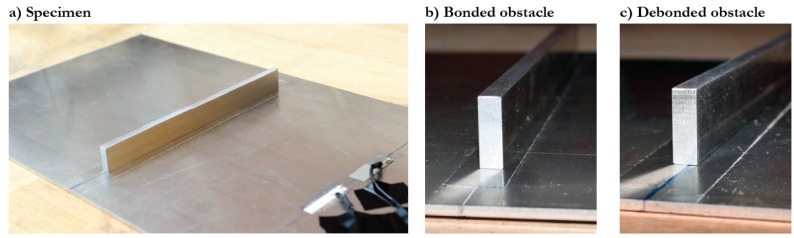
The photography of the specimen.

The input voltage signal

(1) $$p\left(t\right)=V_{0}\frac{1}{2}\cos\left(2\pi ft\right)\left(1-\cos\left(\frac{2\pi ft}{N_{c}}\right)\right),\;0<t<\frac{N_{c}}{f},$$

with a central frequency *f* and $N_{c}=5$ cycles, was applied to the PWATs, and the velocities of the excited Lamb waves were measured at the surface of the plate with a 3D laser Doppler vibrometer from Polytec. Photographs of the experimental specimen are presented in Figure 2, where the usage of the epoxy film to create bonded and debonded contact conditions between the obstacles and the plate is clearly visible.

## 3. Two-Dimensional Mathematical Model

### 3.1. Governing and Constitutive Equations

At first, a brief description of the mathematical formulations that will be employed is given. The considered problem is treated by taking into account the plain strain assumption. The material properties are specified by the tensor of elastic constants $C_{ijkl}$ and the mass density ρ, while the piezoelectric material is also characterized by the piezoelectric and dielectric constants $e_{kij}$ and $\varepsilon_{ik}$, respectively. The constitutive equations in the general case of piezoelectric material are written as follows:

(2) $$\begin{array}{c} \sigma_{ij}=C_{ijkl}s_{kl}-e_{kij}E_{k},\;D_{i}=e_{ikl}s_{kl}+\varepsilon_{ij}E_{j}, \end{array}$$

where $\sigma_{ij}$ are components of the stress tensor, $D_{i}$ are components of the electric displacement vector, $s_{kl}=\frac{1}{2}\left(u_{k,l}+u_{l,k}\right)$ is the strain tensor, expressed in terms of derivatives $u_{k,l}$ of mechanical displacements $u_{k}$ with respect to $x_{l}$, and $E_{k}=-\varphi_{,k}$ are the components of the electric field, expressed in terms of the electric potential φ. For elastic media, the electric part ($E_{k}$) is omitted in (2) and elastic constants $C_{ijkl}$ are expressed in terms of the Lame constants λ and μ (for more details see, e.g., [39]).

Therefore, the governing equations

(3) $$\begin{array}{c} \sigma_{ij,j}=\rho \frac{\partial^{2}u_{i}}{\partial t^{2}} \end{array}$$

are employed to describe the dynamics of the elastic and piezoelectric materials, whereas the relations

(4) $$\begin{array}{c} D_{i,i}=0 \end{array}$$

are valid for piezoelectric materials only, i.e., for PWATs. In the case of elastic material, Equation (3) can be simplified to the Lame equations:

(5) $$\left(\lambda +\mu \right)\nabla \mathrm{div}u\left(x,t\right)+\mu ▵u\left(x,t\right)-\rho \frac{\partial^{2}u\left(x,t\right)}{\partial t^{2}}=0.$$

### 3.2. Mathematical Formulation of the Problem with a PWAT and Two Obstacles

**Figure 3:**
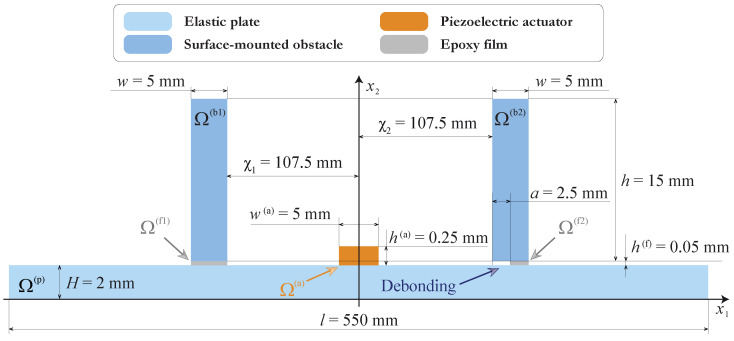
The geometry for the boundary value problem corresponding to the experiment.

Let us consider a two-dimensional mathematical model that allows us to describe the experiment given in the previous section. The geometry of the problem is demonstrated in Figure 3. An elastic layer of thickness H=2 mm occupies the domain $\Omega^{\left(p\right)}=\left\{\right|x_{1}|<l/2$, $0\leq x_{2}\leq H\}$, and a PWAT attached at the surface of the waveguide $\Omega^{\left(p\right)}$ and is operating like an actuator that is assumed to occupy the rectangular domain $\Omega^{\left(a\right)}=\left\{\right|x_{1}\left|<w^{\left(a\right)}/2,0\leq x_{2}-H\leq h^{\left(a\right)}\right\}$ of thickness $h^{\left(a\right)}$ and width $w^{\left(a\right)}$. Two elastic rectangular blocks of thickness *h* and width *w* are attached at the surface of the waveguide using $h^{\left(f\right)}=0.05$ mm thick epoxy tape. In the model, the left obstacle $\Omega^{\left(b1\right)}=\left\{-w\leq x_{1}+\chi_{1}\leq 0,0\leq x_{2}-H-h^{\left(f\right)}\leq h\right\}$ is fully bonded with the waveguide via the film $\Omega^{\left(f1\right)}=\left\{-w\leq x_{1}+\chi_{1}\leq 0,0\leq x_{2}-H\leq h^{\left(f\right)}\right\}$, whereas the right obstacle $\Omega^{\left(b2\right)}=\left\{0\leq x_{1}-\chi_{2}\leq w,0\leq x_{2}-H-h^{\left(f\right)}\leq h\right\}$ is partially debonded and the epoxy film occupies the domain $\Omega^{\left(f2\right)}=\left\{a\leq x_{1}-\chi_{1}\leq w,0\leq x_{2}-H\leq h^{\left(f\right)}\right\}$.

The lower boundary of the PWAT is grounded, and the side boundaries are charge-free:

(6) $$\varphi =0,\;x\in \Omega^{\left(a\right)}\cap \Omega^{\left(p\right)}$$

(7) $$D_{1}=0,\;x\in \partial \Omega^{\left(a\right)}\backslash \left(x_{2}=\left\{H,H+h^{\left(a\right)}\right\}\right).$$

An electric potential p(t) is applied on the upper boundary of the PWAT

(8) $$\varphi =p\left(t\right),\;x\in \Omega^{\left(a\right)}\cap \left(x_{2}=H+h^{\left(a\right)}\right)$$

to excite guided waves in the waveguide $\Omega^{\left(p\right)}$. All outer boundaries ∂Ω of the whole structure Ω are assumed to be stress-free:

(9) $$\begin{array}{c} \sigma_{ij}\cdot n_{j}=0,\;x\in \partial \Omega . \end{array}$$

Here, n is the normal vector. For convenience, the traction vector τ = $\left\{\sigma_{12},\sigma_{22}\right\}$ composed of tangential and normal stresses is introduced. The continuity of stresses and displacements is assumed in the contact areas between the surface-mounted objects and the waveguide, i.e.,

(10) $$\begin{array}{c} \left[u\right]=\left[\tau \right]=0,\;x\in \left(\Omega^{\left(p\right)}\cap \left(\Omega^{\left(f1\right)}\cup \Omega^{\left(f2\right)}\cup \Omega^{\left(a\right)}\right)\right)\cup \left(\Omega^{\left(f1\right)}\cap \Omega^{\left(b1\right)}\right)\cup \left(\Omega^{\left(f2\right)}\cap \Omega^{\left(b2\right)}\right). \end{array}$$

Here, square brackets [f] denote the jump of a given function f.

### 3.3. Experimental Verification of the Mathematical Model

To validate the obtained mathematical model, a comparison with the experimental measurements in the case of the thin PWAT with dimensions 5×30×0.25 mm was performed. The standard FEM software “Comsol Multiphysics” was used to solve the mathematical problem with the following dimensions of the actuator ($h^{\left(a\right)}=0.25$ mm, $w^{\left(a\right)}=5$ mm), the obstacles (h=15 mm, w=5 mm, $\chi_{1}=\chi_{2}=107.5$ mm), and the plate (l=275 mm, H=2 mm). The material properties used for the simulations are given in Table 1.

**Figure 4:**
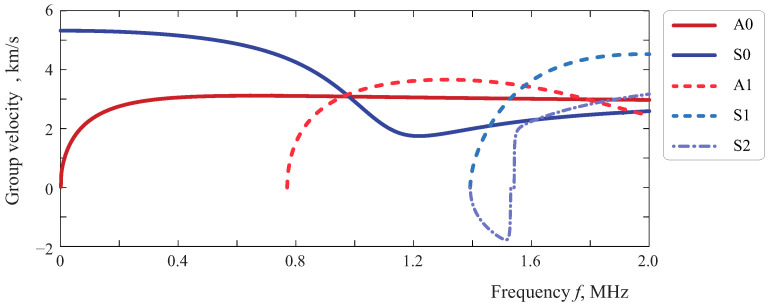
Group velocities of Lamb waves in a 2 mm thickness aluminum plate.

For better interpretation of the results presented below, e.g., time of flights of the incident and scattered Lamb waves, the group velocities of non-attenuating Lamb waves in a 2 mm thickness aluminum plate—predicted theoretically—are depicted in Figure 4.

**Figure 5:**
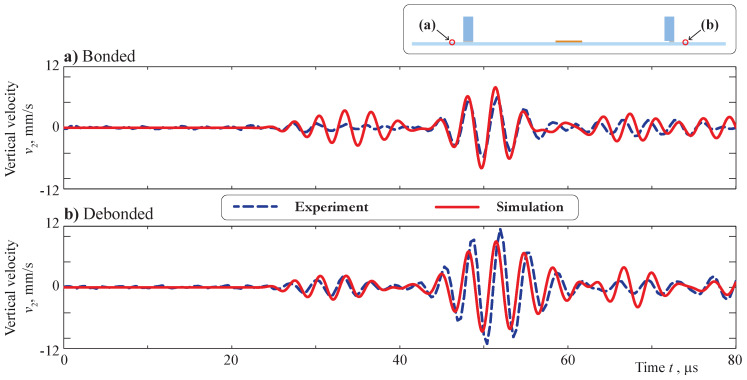
Vertical velocities at the surface of the plate for bonded (**a**) and debonded (**b**) obstacles, which were measured and calculated at central frequency f=300 kHz at the points $x_{1}=\pm 130$ mm.

The velocities of vertical ($v_{2}=u_{2,t}$) and horizontal ($v_{1}=u_{1,t}$) motion measured on the back side of the plate were compared with the transient signal calculated within the employed FEM model. The points $x_{1}=\pm 130$ mm were specified for comparison. The comparison of the vertical velocities $v_{2}$ measured and calculated at a central frequency f=300 kHz is demonstrated in Figure 5. A good agreement of the signals is clearly visible for both bonded and debonded obstacles. In the case of debonding, a small phase shift can be distinguished due to deviation of the real measurement point from nominal $x_{1}=130$ mm from the PWAT’s center.

**Figure 6:**
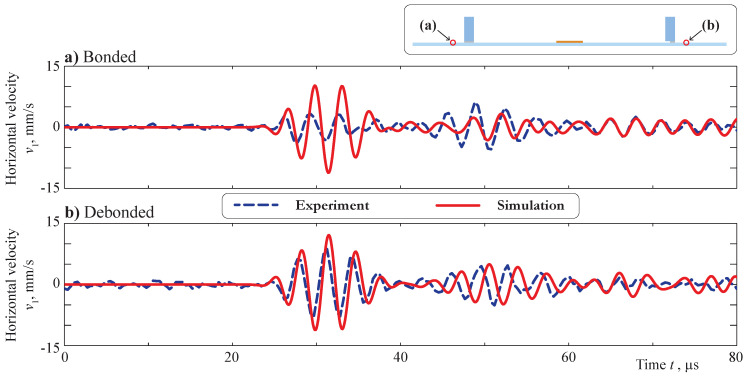
Horizontal velocities at the surface of the plate for bonded (**a**) and debonded (**b**) obstacles measured and calculated at the central frequency f=300 kHz at the points $x_{1}=\pm 130$ mm.

Debonding of the obstacle leads to a growth of the amplitudes and a phase shift; the same effect was predicted by the mathematical model. Still, a discrepancy in the first part of the signals could be noticed, especially in the point $x_{1}=-130$ mm, and the same effect is visible in Figure 6, where horizontal velocities of the motion $v_{1}$ measured and calculated with a central frequency f=300 kHz are presented. Again, debonding of the obstacle results in the amplitudes’ growth for both experimental and simulation signals.

**Figure 7:**
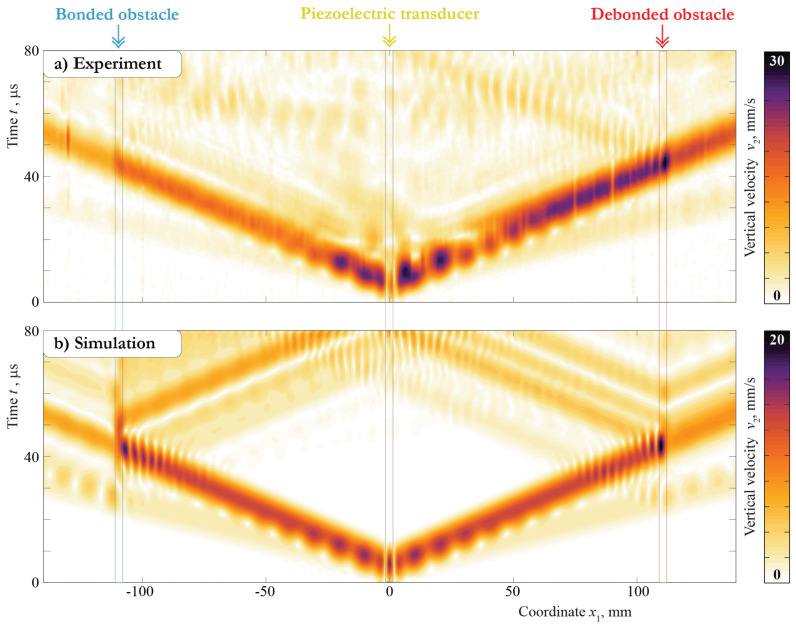
Vertical velocities at the surface of the plate for bonded (**a**) and debonded (**b**) obstacles measured and calculated with the central frequency f=300 kHz.

**Figure 8:**
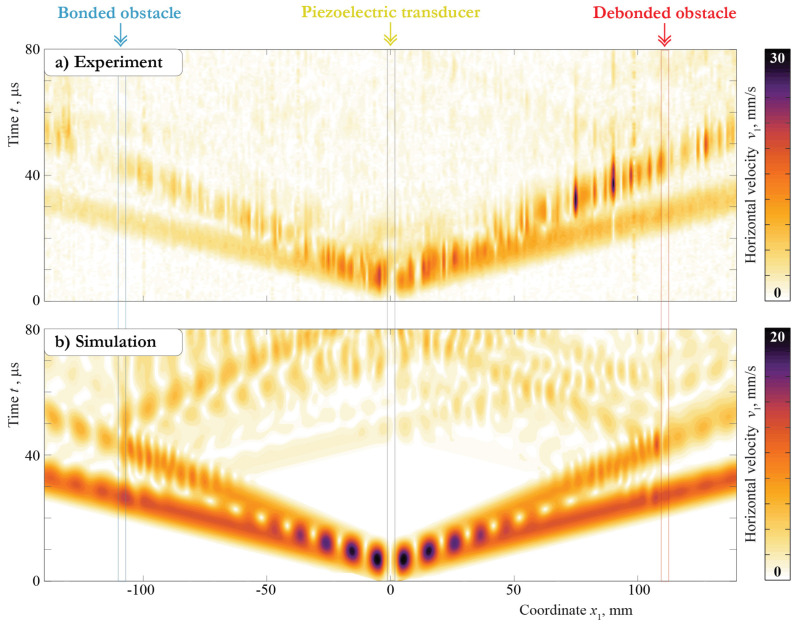
Horizontal velocities at the surface of the plate for bonded (**a**) and debonded (**b**) obstacles measured and calculated with the central frequency f=300 kHz.

To investigate the differences inside the first 80 μs of the measured and simulated signals, the complete wave patterns are presented in Figure 7 for the vertical component and in Figure 8 for the horizontal component measured and calculated at the central frequency f=300 kHz. These plots illustrate the surfaces of the Hilbert transform of the velocities of the motion $v(x_{1},0,0,t)$ measured in the experiment and calculated with the 2D model with dependence on the $x_{1}$ coordinate and time *t*. The propagation of the S0 and A0 Lamb wave modes is clearly visible for both the experimental and simulation signals. In the case of the vertical component, the amplitudes of the A0 mode are higher compared with those of the S0 mode.

At the same time, for the horizontal component, the amplitudes of symmetric and antisymmetric modes are at least of the same value. Moreover, the 2D model provides a stronger excitation of the S0 mode than is visible in the experiment. This effect leads to the discrepancy in the first part of the A-scans, which are depicted in Figure 5 and Figure 6. Both experiments also show that the piezoelectric transducer does not show a perfect symmetric actuation, as the signals are also not symmetric for locations x≤107.5 mm. This is possibly caused by a non-symmetric bonding layer thickness and was not taken into account for the modeling, leading to additional discrepancies. The soldering points’ orientations and the cables attached at the plate’s face can cause non-symmetric wave excitation as well. Still, the behavior of the measured and simulated wavefronts is the same, and, more importantly, the effect of the obstacle debonding is correctly predicted by the model. Such comparison results allow further analysis of the Lamb wave scattering due to debonding in the contact zone between the surface-mounted obstacle and the plate-like structure by means of the obtained 2D model.

## 4. Analysis: Wave Phenomena

### 4.1. Mathematical Formulation of the Problem for the Incidence of a Selected Lamb Wave

**Figure 9:**
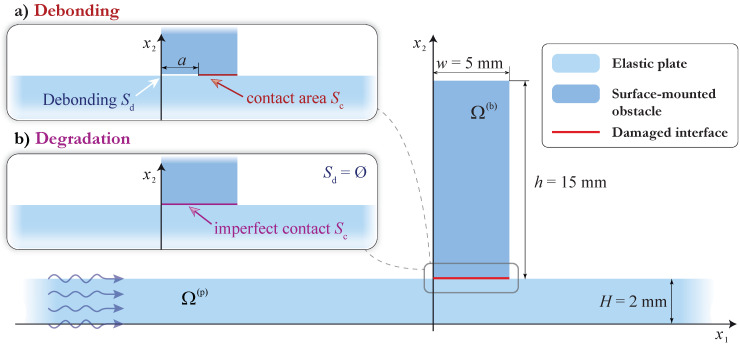
The geometry for the mathematical model.

To investigate interaction of guided waves with the surface-mounted block with interface defects, the in-plane harmonic steady-state motion with the angular frequency ω=2πf is considered. An elastic rectangular block $\Omega^{\left(b\right)}=\left\{0\leq x_{1}\leq w,0\leq x_{2}-H\leq h\right\}$ with dimensions w×h mm is attached at the surface of the infinite elastic layer $\Omega^{\left(p\right)}=\left\{\right|x_{1}\left|<\infty ,0\leq x_{2}\leq H\right\}$ mm, as shown in Figure 9. Correspondingly, displacements in the elastic waveguide and in the elastic obstacle satisfy the Lame equations obtained from (3) after applying the Fourier transform with respect to *t*:

(11) $$\left(\lambda +\mu \right)\nabla \mathrm{div}u\left(x\right)+\mu ▵u\left(x\right)+\rho \omega^{2}u\left(x\right)=0.$$

Two kinds of damaged interfaces $S=\Omega^{\left(b\right)}\cap \Omega^{\left(p\right)}$ between the waveguide and the obstacle are considered in this section: debonding at the interface and degradation of the interface. In the case of debonding, a crack of width *a* at the interface *S* is assumed so that the interface $S=S_{d}\cup S_{c}$ consists of the debonded area $S_{d}$, where the stress-free boundary conditions (9) are assumed, and contact area $S_{c}$, where wave-fields satisfy continuous boundary conditions (10). For degradation modeling, i.e., for the the second kind of damage, the spring boundary condition [36,37]

(12) [τ]=0,τ=S[u],x,∈S

with diagonal stiffness matrix S=diag(κ,κ), is introduced. SBC (12) assumes the continuity of the traction vector at the interface, where the displacement vector has a jump proportional to the traction vector. In both cases, the outer surfaces of the waveguide and the obstacle are stress-free. The mathematical model was constructed with the following dimensions of the obstacle (h=15 mm, w=5 mm) and the plate (H=2 mm), while the material properties are given in Table 1.

The stated problem is solved with the SAHA, which is more suitable for the problem of sole guided wave scattering [17]. In this case, the displacement field $u^{\left(\mathrm{in}\right)}\left(x\right)$ of the Lamb wave with wavenumber ζ incoming from −∞ (i.e., propagating from the left to the right according to the system of coordinates shown in Figure 9) is calculated via Cauchy’s residue theorem as follows:

(13) $$\begin{array}{c} u^{\left(\mathrm{in}\right)}\left(x\right)=-i\;\mathrm{res}K\left(\alpha ,x_{2}\right)Q\left(\alpha \right){|}_{\alpha =-\zeta }\cdot e^{i\zeta x_{1}}. \end{array}$$

Here, $K(\alpha ,x_{2})$ and Q(α) are the Fourier transform of the Green’s matrix and the arbitrary surface load function (more details can be found in [31]).

The hybrid mathematical approach allows the investigation of the elastic wave energy transfer from the source into the waveguide and the amount of the elastic wave energy carried by each Lamb wave. This analysis is based on the time-averaged power density vector e(x), or Umov–Poynting vector:

(14) ej=ω2Imσ1ju1*+σ2ju2*.

The defective contact between the obstacle and the waveguide results in a partial reflection of the guided waves, and therefore, it is convenient to calculate the transmission and reflection coefficients, which is possible within the SAHA. The wave energy distribution coefficients $\beta_{m}^{\pm }$ (*m* corresponds to a Lamb wave) are introduced via the integration of the horizontal component $e_{1}$ of the time-averaged power density vector along a certain cross-section of the waveguide in a far-field zone. The energy distribution coefficients

(15) $$\begin{array}{c} \beta_{m}^{\pm }=P_{m}^{\pm }/P^{0} \end{array}$$

are the ratios between the wave energy flux $P_{m}^{\pm }$ carried by *m*-th Lamb wave in directions $x_{1}\to \pm \infty$ and the wave energy transmitted by a certain incoming Lamb wave mode

(16) $$\begin{array}{c} P^{0}=\int_{0}^{H}e_{1}^{\left(\mathrm{in}\right)}\left(x_{1},x_{2}\right)dx_{2}. \end{array}$$

To extract wave-fields related to scattered Lamb waves with a specific wavenumber ζ corresponding to a specific Lamb wave, relations similar to Equation (13) are employed (for more details, see [17,31]). It should be mentioned that the equality

(17) $$\begin{array}{c} \sum_{m}\left(\beta_{m}^{+}+\beta_{m}^{-}\right)=1. \end{array}$$

is valid for energy distribution coefficients.

### 4.2. Debonding between the Obstacle and the Plate

To construct an automatic SHM algorithm for damage detection, it is essential to gain knowledge on how a surface-mounted obstacle scatters guided waves, including partial debonding and degradation of the contact. In this subsection, the effect of the debonding (infinitesimally thin crack between the obstacle and the waveguide) is investigated (see Figure 9a, where a graphical representation of the mathematical problem is shown). Two cases of one-sided debondings were considered: $S_{d}=\left\{0\leq x_{1}\leq a,x_{2}=H\right\}$ and $S_{d}=\left\{a\leq x_{1}\leq w,x_{2}=H\right\}$. It was revealed that the differences in the resulting wave-fields are small. Therefore, the results for debonding from only the left side are presented here ($S_{d}=\left\{0\leq x_{1}\leq a,x_{2}=H\right\}$), and the debonding rate is calculated as a/w·100%.

To investigate the interaction of antisymmetric and symmetric Lamb waves with the obstacle at various central frequencies, the propagation of incident A0 and S0 Lamb waves is modeled. Coefficients $\beta_{m}^{\pm }$ are used in accordance with (15) to investigate how much energy of the incident wave is transmitted, reflected, or converted depending on the frequency *f*.

**Figure 10:**
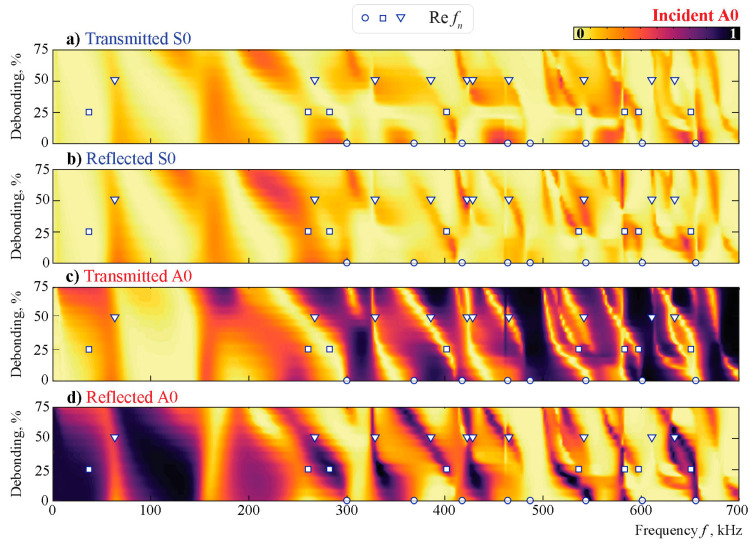
The transmission and reflection coefficients $\beta_{m}^{\pm }\left(f,a\right)$ for the incident A0 mode scattered by a debonded obstacle and the eigenfrequencies calculated for three different debonding states.

**Figure 11:**
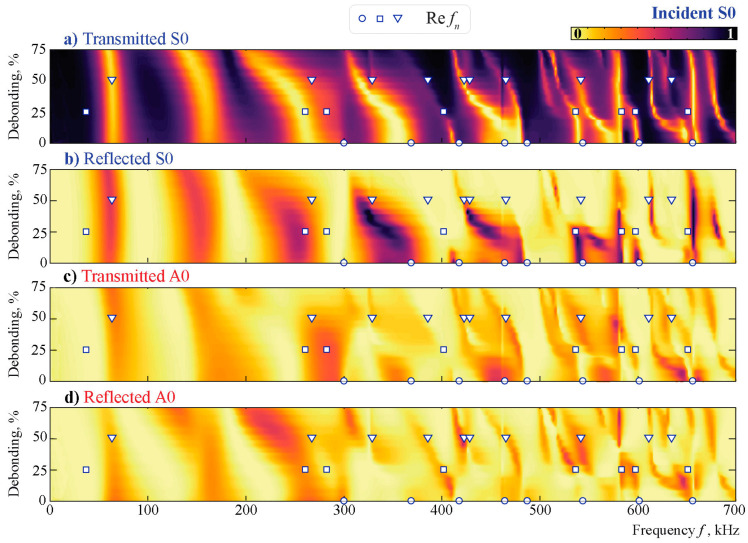
The transmission and reflection coefficients $\beta_{m}^{\pm }\left(f,a\right)$ for the incident S0 mode scattered by a debonded obstacle and the eigenfrequencies calculated for three different debonding states.

Figure 10 and Figure 11 illustrate the energy distribution coefficients $\beta_{m}^{\pm }\left(f,a\right)$ given by relation (15) depending on the frequency and debonding parameter when the m= A0 or m= S0 Lamb wave is excited. Coefficients $\beta_{m}^{\pm }$ indicate the share of the *m*-th mode in the wave energy flow $P^{\pm }$, which is part of the induced energy flux $P^{0}$ propagating in directions $x_{1}\to \pm \infty$. The effect of the debonding presence is minimal at the lower frequencies (f<150 kHz). Little influence is detected in the frequency range 150<f<350 kHz, and only with the higher frequencies (f>350 kHz) does the debonding have perceptible influence. Three frequency ranges are visible when the A0 mode is incident: where A0 is mostly reflected by the obstacle (f<150 kHz), where approximately 50% of the wave energy is transmitted and reflected (150<f<300 kHz), and when transmission zones frequently change by the reflection zones, depending highly on the frequency and less highly on the debonding size (f>300 kHz). The uncommonly high reflection of the A0 mode at lower frequencies is studied further. The conversion rate of the S0 mode is also low, but unlike the antisymmetric mode, S0 is reflected only partly and only inside narrow frequency ranges. When the block is severely debonded (a/w>50%), the S0 mode’s reflection from the obstacle is minimal and strongly dependent on the frequency *f*. Thus, it can be concluded that the A0 mode is scattered from the obstacle mostly with the lower frequencies (f<300 kHz), while the S0 mode interacts with the surface-mounted obstacle only in specific frequency ranges and passes through for the frequencies outside these ranges, which are in the large majority.

The eigenfrequencies $f_{n}$ of the unbounded aluminum layer with the debonded block of dimensions w×h, as shown in Figure 9, are marked in Figure 10 and Figure 11 by circles, squares, and triangles for debonding rates of 0%, 25%, and 50%, respectively. The eigenfrequencies independently calculated using the SAHA and the FEM are given in Table 2. The two numerical methods applied in this study are in a good agreement. One can also see that the eigenfrequencies are situated relatively close to the real axis, and some peaks in the $\beta_{m}^{\pm }\left(f\right)$ plots correspond to the real values of the eigenfrequencies. Nevertheless, a strong influence of eigenfrequencies on Lamb wave conversion and reflection cannot be reported.

**Figure 12:**
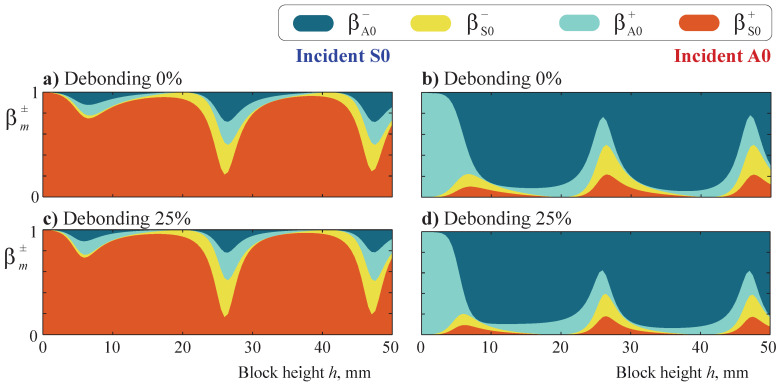
The transmission and reflection coefficients $\beta_{m}^{\pm }\left(h\right)$ for the incident S0 (**a**,**c**) and A0 (**b**,**d**) modes scattered by the obstacle of height *h* and width w=5 mm at frequency f=25 kHz with a debonding rate of 0% at a=0 (**a**,**b**) and a debonding rate of 25% at a=1.25 mm (**c**,**d**).

The effect of the variation of the width of a rectangular block on Lamb wave transmission was analyzed in [17]. To investigate an uncommon A0 reflection at lower frequencies and the influence of the block’s height, the transmission and reflection of fundamental Lamb waves were analyzed with respect to the block’s height *h*. Figure 12 shows the transmission and reflection coefficients $\beta_{m}^{\pm }\left(h\right)$ for incident S0 and A0 modes for a bonded and 25% debonded block of width w=5 mm at frequency f=25 kHz. For both fundamental Lamb waves, one can see periodically situated peaks. It is also clearly seen that the A0 mode is almost fully transmitted if the height h<5 mm, while a strong reflection is observed if the height increases. The effect can be explained by the properties of the A0 mode, which has a dominating vertical component (such a strong reflection is not observed for the S0 mode).

**Figure 13:**
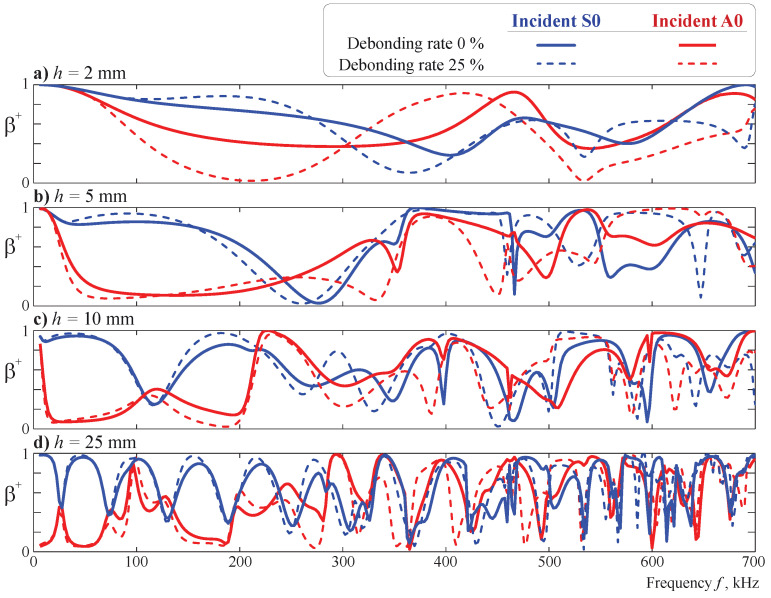
The total transmission coefficient $\beta^{+}\left(f\right)$ for the incident S0 and A0 modes scattered by the obstacle of height h=2 mm (**a**), 5 mm (**b**), 10 mm (**c**), or 25 mm (**d**) and width w=5 mm, as well as a debonding rate of 0% with a=0 or a debonding rate of 25% with a=1.25 mm.

Figure 13 depicts the total transmission coefficient

$$\beta^{+}=\sum_{m}\beta_{m}^{+}$$

for the obstacles of width w=5 mm and four different heights: h=2,5,10,25 mm. Height increase leads to a more complicated distribution of the transmission coefficient because the number of eigenfrequencies in a certain frequency range increases and the corresponding eigenforms become more complex. The difference between the transmission coefficients for bonded and debonded obstacles is more pronounced for smaller heights (cf. Figure 13a–d). The latter can be explained by the eigenforms of the waveguide with an obstacle: wave localization in the upper part of the obstacle is more similar for bonded and debonded states if the rectangular block is taller because the height of the obstacle has a somewhat higher influence on wave propagation than the debonding size. The resonance peaks visible in the transmission coefficient plots coincide for both incident A0 and S0 modes. This effect is noticeable for the four calculated heights of the obstacle.

### 4.3. Adhesive Degradation between the Obstacle and the Plate (Imperfect Contact)

**Figure 14:**
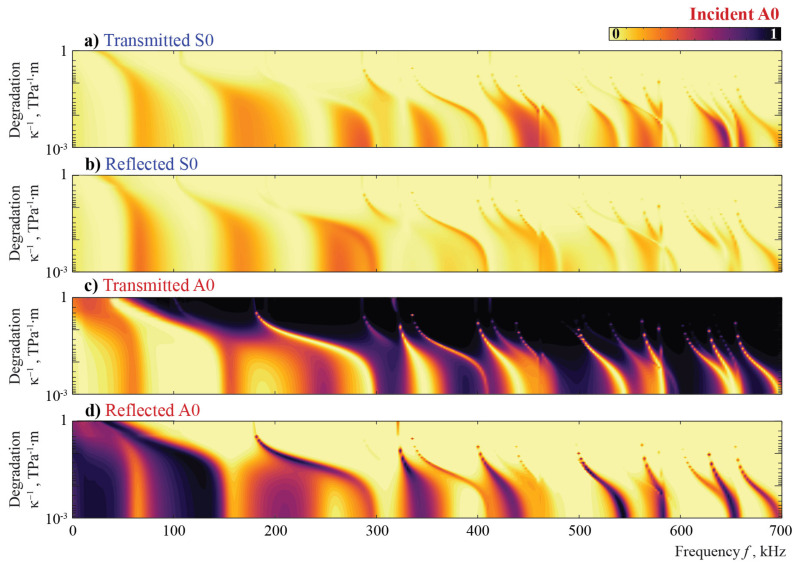
The transmission and reflection coefficients $\beta_{m}^{\pm }\left(f,\kappa^{-1}\right)$ for the incident A0 mode scattered by an obstacle with a degradation.

**Figure 15:**
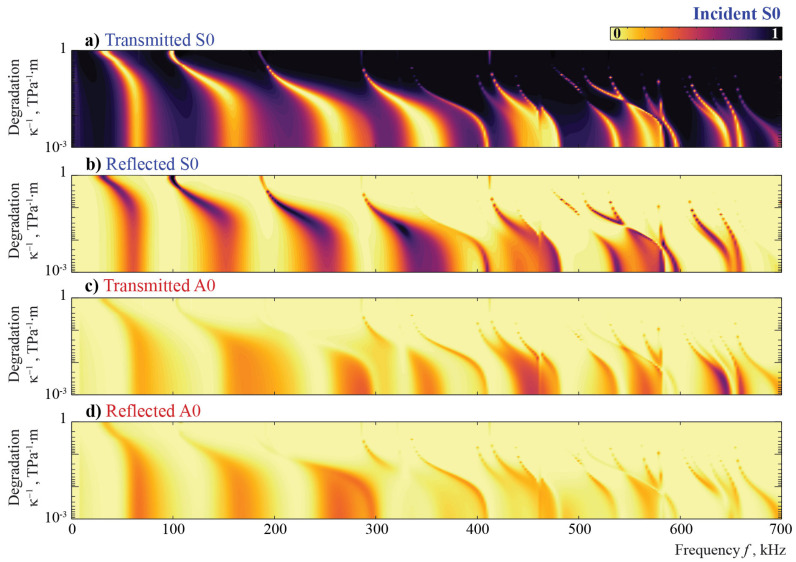
The transmission and reflection coefficients $\beta_{m}^{\pm }\left(f,\kappa^{-1}\right)$ for the incident S0 mode scattered by an obstacle with a degradation.

Another widespread type of defect that is investigated in this study is the degradation of the adhesive or contact between a surface-mounted obstacle and a waveguide (see Figure 9b). This kind of damage is modeled here with the spring boundary condition (12), where the variation of the component κ of the stiffness matrix S allows one to change the imperfectness of the contact between the plate and the obstacle. Figure 14 and Figure 15 illustrate the energy distribution coefficients $\beta_{m}^{\pm }$ with respect to frequency *f* and stiffness κ. Since larger values of $\kappa^{-1}>>1$ correspond to an almost fully debonded obstacle and $\kappa^{-1}=0$ describes perfect contact at the interface, $\kappa^{-1}$ is used in the further analysis as a degradation parameter to obtain a parameter describing the severity of damage in a similar manner to that of the debonding case considered in the previous subsection.

Once again, it is observed that the A0 mode is fully reflected from the obstacle at lower frequencies (f<100 kHz). One can see narrow peaks corresponding to local resonances; these peaks depend on the stiffness κ and they almost disappear if $\kappa <10^{13}$ TPa·m${}^{-1}$ at frequencies f>150 kHz. Inside the frequency range 100<f<300 kHz, the reflection portion depends on the frequency and stiffness κ, while for higher frequencies (f>300 kHz), the reflection depends only on the frequency, and the A0 mode passes through the obstacle without any reflection or conversion for stiffness $\kappa <10^{13}$ TPa·m${}^{-1}$.

Surfaces $\beta_{m}^{\pm }\left(f,\kappa^{-1}\right)$ for S0 scattering are similar to the case of A0. Though most of the resonance peaks are revealed in the same frequency and stiffness ranges, some of them are distinct for the two considered Lamb waves. With the frequency growth, the degradation has a stronger influence on the energy distribution coefficients. For lower frequencies, the S0 mode is reflected by the obstacle, even for highly deteriorated contact $\kappa =10^{12}$ TPa·m${}^{-1}$, e.g., f=30 kHz. For both fundamental Lamb waves, the noticeable effect of conversion is visible only for higher frequencies and specific values of κ.

## 5. Analysis: Damage Detection

### 5.1. Mathematical Formulation of the Problem

**Figure 16:**
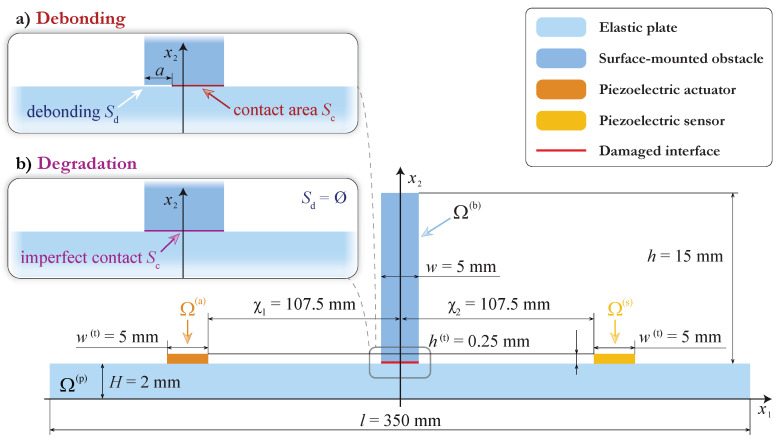
The geometry for the mathematical model.

To investigate the influence of debonding of the surface-mounted obstacle on the sensor voltage signal, a finite-length elastic plate with a block and two PWATs operating like an actuator and a sensor is modeled. Thus, an elastic rectangular block $\Omega^{\left(b\right)}=\left\{\right|x_{1}|\leq w/2$, $0\leq x_{2}-H\leq h\}$ with dimensions w×h mm and two piezoelectric transducers $\Omega^{\left(a\right)}$, $\Omega^{\left(s\right)}$ with dimensions $w^{\left(t\right)}=5$ mm and $h^{\left(t\right)}$ = 0.25 mm are attached at the surface of the elastic plate $\Omega^{\left(p\right)}=\left\{\right|x_{1}\left|<l/2\right\},\left\{0\leq x_{2}\leq H\right\}$ mm (see Figure 16). Two PWATs $\Omega^{\left(a\right)}=\{0\leq x_{1}+\chi_{1}\leq w^{\left(t\right)}$, $0\leq x_{2}-H\leq h^{\left(t\right)}\}$ and $\Omega^{\left(s\right)}=\{0\leq x_{1}-\chi_{2}\leq w^{\left(t\right)}$, $0\leq x_{2}-H\leq h^{\left(t\right)}\}$, situated at the same distance from the obstacle, operate as an actuator and as a sensor, respectively.

The governing Equations (3) and (4) are employed for simulating wave motion in elastic and piezoelectric domains. The same two kinds of defects as in the previous section are considered: debonding and degradation. Stress-free boundary condition (9) is chosen for the outer surfaces of the waveguide, two PWATs, and the obstacle, as well as for the delaminated area $S_{d}$ in the case of debonding. The continuity boundary condition (10) is assumed in the contact area between the PWATs and the waveguide and at the internal boundary $S_{c}$. Two kinds of defects at the interface $S=\Omega^{\left(b\right)}\cap \Omega^{\left(p\right)}$ between the waveguide $\Omega^{\left(p\right)}$ and the obstacle $\Omega^{\left(b\right)}$ are considered in this section: a debonding at the interface and a degradation of the interface. In the case of debonding, a crack of width *a* at the interface *S* is assumed so that the interface $S=S_{d}\cup S_{c}$ consists of the debonded area $S_{d}$, where the stress-free boundary condition (9) is assumed, and the contact area $S_{c}$, where wave-fields satisfy continuous boundary condition (10). For the degradation case, spring boundary condition (12) is used again.

Boundary conditions (6) and (7) are valid for both PWATs: The side boundaries of the transducers are free of charge and the lower boundaries of both transducers are grounded. In the case of the actuator $\Omega^{\left(a\right)}$, the electric potential applied on the upper surface is simulated via (8). To simulate the sensor $\Omega^{\left(s\right)}$, an unknown electric potential φ(x,t) is introduced at the upper surface of the sensor $S^{\left(\phi \right)}=\Omega^{\left(s\right)}\cap \left(x_{2}=H+h^{\left(t\right)}\right)$, i.e.,

(18) $$\varphi^{\left(s\right)}\left(x,t\right)=\phi \left(t\right),\;x\in S^{\left(\phi \right)}.$$

In addition to (18), electric charge is also assumed to be equal to zero on the upper surface $S^{\left(\phi \right)}$ of a sensor:

(19) $$\int_{S^{\left(\phi \right)}}D_{2}dx_{1}=0.$$

The simulation was performed for the following geometrical parameters of the two PWATs ($h^{\left(t\right)}=0.25$ mm, $w^{\left(t\right)}=5$ mm, χ=112.5 mm), the obstacle (h=15 mm, w=5 mm), and the plate (l=175 mm, H=2 mm) with voltage $V_{0}=70$ V; the material properties are given in Table 1.

### 5.2. Transient Signals and Damage Indices

When using Lamb waves for automated damage detection with permanently installed PWATs, not only the usage of actuator and sensor, but also the usage of a short signal with a narrow frequency band is preferable for simplifying data analysis. This way, the effect of the reflections does not overlay with the effect of possible damage easily, and the signal still does not have high dispersion, which complicates physics-based data analysis. Both factors are contradictory; therefore, a trade-off between the length of the signal and width of the frequency band has to be realized. A Hann-windowed *N*-cycle toneburst was proven to be suitable for many applications decades ago [11] and is used in industrial applications of guided waves. At the same time, the signal’s central frequency needs to be chosen in such a way that the excited wave interacts with the damage, which should be found with the automated damage detection system based on guided waves. If this frequency is not perfectly known in advance, the usage of rectangular pulses that include a wide frequency spectrum has been mentioned recently as an alternative to using the joint analysis of several frequencies if statistical data analysis is used as a data evaluation strategy.

The chosen signal is not necessarily the best for all methods of automated data evaluation. Within this publication, two very simple feature extraction techniques are used; both are based on statistical data analysis without taking into account the specific physics, such as time of arrival and signal energy. As the focus is on showing the effects on these damage indices (DIs) and not the best choice of DI, simple algorithms that are widely used in the community were chosen. First, the two different signal types are described. Afterwards, the chosen damage indices used for data evaluation are documented.

#### 5.2.1. Input Signals

Two kinds of input signals are used. The first input voltage signal has the form of $N_{c}$ Hann-windowed cycles of the cosine with a central frequency *f* according to relation (1). The second input voltage signal is a rectangular pulse of duration *b* μs

$$p\left(t\right)=\left\{\begin{array}{cc} V_{0}, & 0\leq t\leq b\\ 0, & \mathrm{otherwise}. \end{array}\right..$$

**Figure 17:**
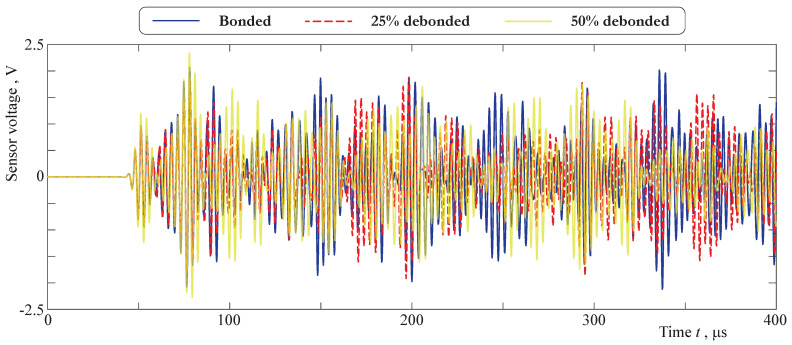
Voltage signals obtained from the sensor for debonding rates of 0%, 25%, and 50%.

An example of the voltage signals obtained from the sensor for debonding rates of 0%, 25%, and 50% is shown in Figure 17. The first parts of the sensor signals (t<90 μs) for all the debonding rates are quite similar, whereas later moments of time.

#### 5.2.2. Damage Indices

Two damage indices are evaluated here for two modeled changes of the setup, i.e., for the debonding of the obstacle and the degradation of the contact between the plate and the obstacle. As a first damage index, the mean root-mean-square value of the differences of the components of two vectors containing the data on the undamaged and the damaged states, $y^{H}$ and $y^{D}$, is used:

$$DI_{\mathrm{RMSM}}=\sum_{i=1}^{M}\sqrt{\frac{\sum {\left(y_{i}^{D}-y_{i}^{H}\right)}^{2}}{\sum {\left(y_{i}^{H}\right)}^{2}}}.$$

Here, vectors $y^{m}=\left\{y_{1}^{m},\ldots ,y_{M}^{m}\right\}$ (m= {H, D}) are composed of the sensor’s transient voltage signals in the moments of time $t_{i}$, (i=1,…,M). This indicator has already been suggested and used in a number of applications, e.g., [12].

As a second damage index, a variation

$$DI_{\mathrm{CCH}}=1-CC$$

is used. It is calculated as the correlation coefficient

$$CC=\frac{V_{12}}{\sqrt{V_{11}V_{22}}}.$$

from the covariance matrix

$$V=\mathrm{cov}\left(H\left[y^{H}\right],H\left[y^{D}\right]\right),$$

which is based on the Hilbert transforms $H[y^{H}]$ and $H[y^{D}]$ of the two signals $y^{H}$ and $y^{D}$, as suggested, e.g., in [10]. Instead of taking the direct time signal, here, the Hilbert transform of the time signal was chosen to decrease the effect of small phase shifts. The effect of this procedure has been shown in [1].

### 5.3. Effects of Obstacle Debonding (Crack between the Obstacle and the Plate)

**Figure 18:**
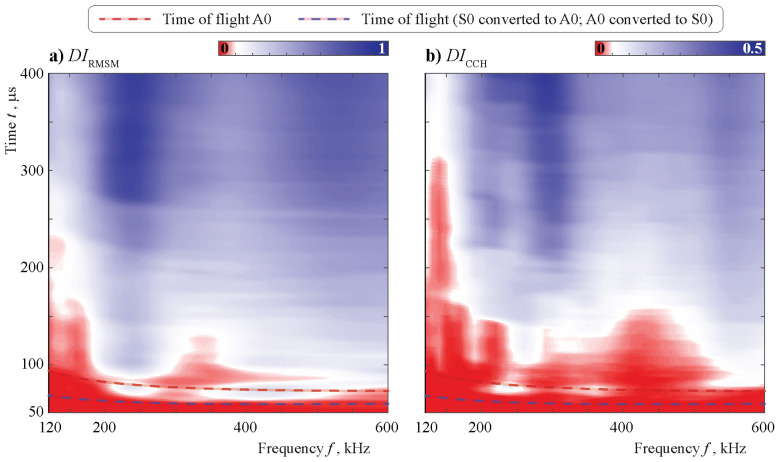
The damage index (DI) $DI_{m}\left(f,t\right)$ calculated using the data from a sensor at different central frequencies *f* in the case of an obstacle with a 25% debonding crack.

**Figure 19:**
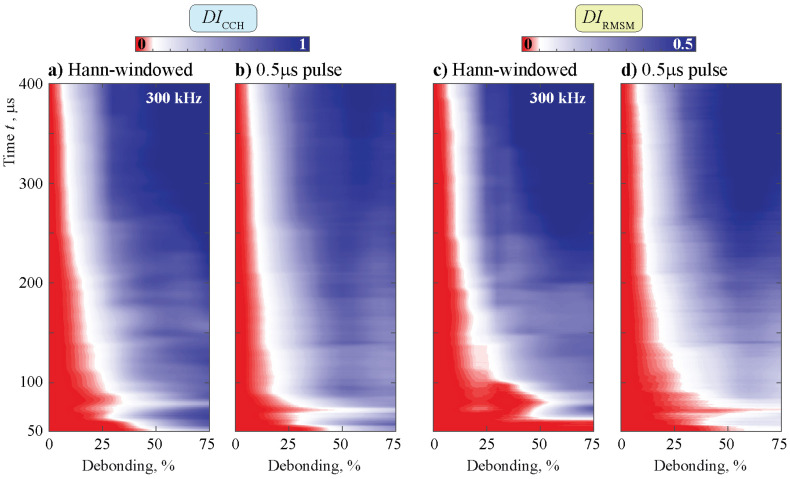
The DI $DI_{m}\left(a/w,t\right)$ calculated using the data from a sensor at different debondings.

**Figure 20:**
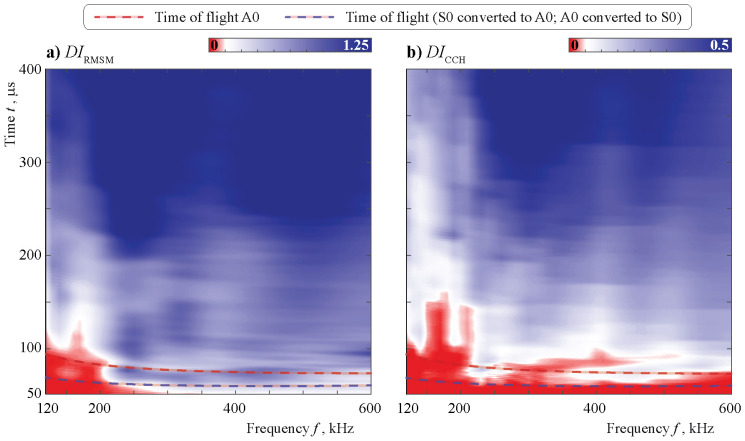
The DI $DI_{m}\left(f,t\right)$ calculated using the data from a sensor at different central frequencies *f* in the case of an obstacle with an imperfect contact stiffness $\kappa =2\times 10^{13}$.

**Figure 21:**
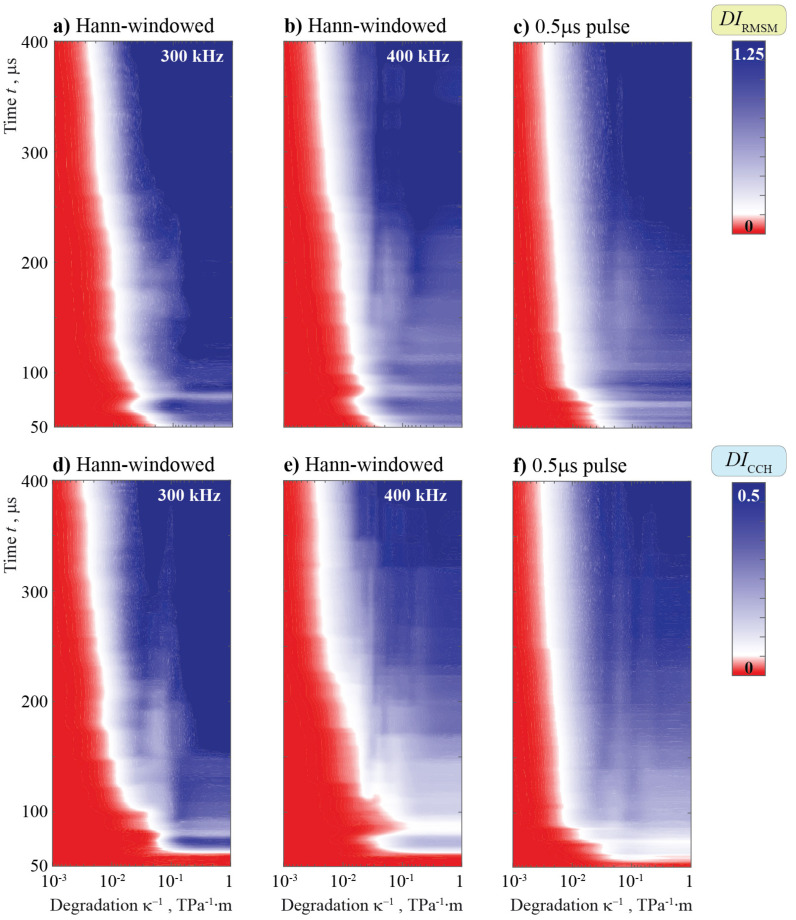
The DI $DI_{m}\left(\kappa^{-1},t\right)$ calculated using the data from a sensor at different imperfect contact stiffnesses κ.

Figure 18, Figure 19, Figure 20 and Figure 21 depict contour plots of the surfaces of two DIs, where the choice of the colormap allowed us to highlight the 10% threshold with white color. It should be noted that different color scales are used for $DI_{\mathrm{CC}}$ and $DI_{\mathrm{RMSM}}$, since their ranges of values are dissimilar. Starting with the effects of the debonding of the obstacle on the two different damage indices for the Hann-windowed signal, it is necessary to take into account the effect of the chosen time window. For 49 central frequencies, equally distributed from 120 to 600 kHz, the chosen time interval was varied from 50 to 400 μs. Both graphs in Figure 18 show that a specific length is necessary before the data analysis will be able to grasp the difference between the signals, which is frequency dependent due to travel time. The times of flights of the incident and scattered Lamb waves calculated using group velocities (see Figure 4) of the A0 and S0 modes in a 2 mm thickness aluminum plate are shown by the dash-dotted curves.

For the interpretation of the data, it is necessary to consider the fact that long signals, which include a lot of reflections, also known as coda-waves [40], have been shown to be sensitive to a variety of influences, and also exhibit larger changes in the “undamaged” state due to small changes in environmental conditions. This ultimately leads to higher thresholds; based on these, a decision about the current state is made. This effect cannot be taken into account in numerical modeling due to the missing influence of environmental conditions and variations in measurement.

Taking into account the results from Figure 10 and Figure 11, which show the same kind of change as in the setup, the frequency influence is now also visible for the chosen damage indices. At around 230 kHz, the results for both DIs are much better than for the 280–300 kHz. The change in the reflected, transmitted, and mode-converted signal in the frequency range of 230 kHz in Figure 10 and Figure 11 is highly dependent on the level of debonding. This is not the case for the frequency range around 290 kHz. Therefore, a detailed analysis of the transmission and reflection coefficients is helpful in explaining the efficiency of automated damage analysis.

Comparing both damage indices, the performance of $DI_{\mathrm{RMSM}}$ is more evenly distributed, and smaller time frames are necessary compared to $DI_{\mathrm{CCH}}$. No specific analysis of the performance is possible due to the already-mentioned fact that the effect of changing environmental conditions was not included in the numerical model, which ultimately defines a possible threshold. As seen in Figure 10 and Figure 11, in the fully bonded stage, the incident A0 and incident S0 waves have already been changed significantly in the higher frequency regime because the block shows high variation with frequency and debonding state.

For the Hann-windowed toneburst at a specific frequency of 300 kHz, as well as for the pulse, the effect of the size of debonding is shown in Figure 19. The plots exhibit the same tendencies for both damage indices and types of input signal. Even with a long signal, a very small debonding is hardly recognizable. While the sensitivity to the time window is comparably low after a specific time for the pulse, it is larger for the Hann-windowed signal. For shorter signals, all combinations show a specific time span, which is unfavorable, leading to decreased sensitivity. The effect is less significant for higher debonding if the Hann-windowed input signal is chosen. For very high rates of debonding, even the very short time is able to detect differences between the two states.

### 5.4. Effects of Adhesive Degradation between the Obstacle and the Plate

As is already visible when comparing Figure 14 and Figure 15 to Figure 10 and Figure 11, the effect of bonding degradation significantly differs from the effect of debonding. This is also visible in the automated data evaluation with the use of damage indices. Both show a better sensing ability in the higher frequency regime. A very interesting phenomenon can be found for $DI_{\mathrm{CCH}}$ at around 400 kHz, as it shows a second decrease in index level after the first increase with increasing time. Clearly, a carefully chosen time interval is of major importance.

For $DI_{\mathrm{CCH}}$, it is not possible for short times to grasp the presence of degradation, even if its severity is high. In general, $DI_{\mathrm{CCH}}$ is only able to detect the change for a time interval larger than approximately 200 μs for f<200 kHz. This is not the case for $DI_{\mathrm{RMSM}}$.

Interestingly, the tendencies that have been seen for the different input signals for the case of debonding in Figure 19 are valid also for the case of degradation in Figure 21. There is a visible tendency of the Hann window in combination with $DI_{\mathrm{RMSM}}$ to give the most continuous signal, i.e., the damage index steadily increases with increasing degradation. Nevertheless, the sensitivity seems to start at slightly greater levels of degradation. Comparing 300 and 400 kHz, it is not only visible that increasing time has no steady trend with respect to $DI_{\mathrm{CCH}}$, but increasing degradation also does not necessary lead to increasing DI, especially a for time of approximately 180 μs. This is not acceptable for many SHM system applications that need a clear trend, at least in the zone of interesting damage size.

When applying this, it will still be important to take into account the levels of the damage indices for the undamaged case, as thresholds are based on these to avoid false calls.

## 6. Conclusions

The analysis of the energy distribution coefficients at the lower frequencies revealed that A0 is strongly reflected by a rectangular 5 × 15 mm${}^{2}$ obstacle, while S0 mostly passes through the obstacle area. However, the effect of the A0 reflection is strongly dependent on the obstacle’s height. For instance, if a 5 × 5 mm${}^{2}$ obstacle is considered, the A0 mode is not reflected by the obstacle at lower frequencies. The debonding of the obstacle influences energy distribution coefficients at rather high frequencies; therefore, an inspection of this kind of damage is more efficient at frequencies f>150 kHz. The effects of debonding and degradation are similar; however, with sever debonding, there are frequencies where Lamb waves are still reflected from the obstacle, even at higher frequencies, while with the high-level degradation of the contact, Lamb waves are fully transmitted without any interaction. From this point of view, it might be more difficult to detect debonding compared to the degradation of the contact. The possibility of damage detection based on damage indices with different input signals and different damage indices was investigated using simulation results. It has been shown that carefully chosen time intervals are of major importance. Both chosen input signals exhibit the same tendencies for both DIs, but the usage of a rectangular pulse enables the user to reduce the time interval due to the wider frequency spectrum in the signal. Of the two simplest DIs chosen for the analysis, $DI_{\mathrm{RMSM}}$ seems to be more preferable.

The results show that the calculation of the reflection and transmission coefficients in the frequency domain enables one to gain knowledge on the sensitivity of specific frequency ranges to the two interface defects analyzed in this publication. The analysis of time-domain data with frequently used input signals shows that the effects are much more smeared and less specific due to the wide frequency spectrum. For the statistics-based damage indicators used, this is also the reason for why the pulse leads to a more steady result, while specific frequencies and time selection for a Hann-windowed input signal can be specifically positive or negative for the sensitivity.

The 2D mathematical model was successfully employed to calculate the energy distribution coefficients, resonance frequencies, transmission, reflection, and conversion rates, as well as sensors’ output signals, for a waveguide with a surface-mounted obstacle with various bonding conditions. The model was verified experimentally prior to implementation of the calculations. However, a 2D model has limits to its application due to the plain strain assumption. Therefore, in future work, the approach is to be enhanced into a 3D model to take into account the orientation of the soldering points, wrapped electrodes of the transducers, oblique incidence, kissing/clapping bonds, and other specific effects.

## Figures and Tables

**Table 1:** Material properties.

Material	Elastic Constants	Piezoelectric Constants	Dielectric Constants	Density
	[GPa]	[${C/m}^{2}$]	$10^{-9}$ [F/m]	[${\mathrm{kg}/m}^{3}$]
Aluminum	λ=51.1	—	—	2700
	μ=26.3			
Epoxy film	λ=0.227	—	—	930
	μ=1.396			
PIC 155	$C_{1111}=120$	$e_{211}=-7.24$	$\varepsilon_{11}=9.12$	7800
	$C_{1112}=67.3$	$e_{212}=13.77$	$\varepsilon_{22}=7.55$	
	$C_{2222}=94.4$	$e_{112}=11.91$		
	$C_{1212}=22.3$			

**Table 2:** Eigenfrequencies (kHz) of an H=2 mm thickness aluminum plate with a rectangular aluminum block debonded on one side (w=5 mm width, h=15 mm height).

Debonding 0%		Debonding 25%		Debonding 50%	
SAHA	FEM	SAHA	FEM	SAHA	FEM
302.4−5.5i	302.3−5.5i	37.8−400.5i		63.6−12.1i	
371.9−18.9i	368.7−17.4i	262.6−26.5i		265.6−22.1i	268.3−15.6i
416.9−2.1i	416.9−2.1i	284.2−202.2i		316.5−8.9i	331.8−1.9i
467.5−0.1i	467.5−0.1i	404.8−6.9i	406.6−6.5i	387.1−8.8i	387.9−8.3i
489.7−3.3i	489.4−3.3i	541.7−4.3i	542.3−3.9i	425.3−6.9i	425.3−6.4i
546.8−4.7i	546.8−4.7i	587.2−1.7i	589.3−1.5i	468.6−0.9i	467.3−0.9i
607.6−3.0i	607.6−3.0i	602.3−4.9i	603.2−4.3i	545.1−8.7i	545.1−8.0i
659.5−4.4i	659.5−4.4i	674.1−1.8i	665.4−1.8i	616.6−7.0i	623.4−1.0i
				638.5−3.8i	638.2−3.8i

## Data Availability

Not applicable.

## Abbreviations

The following abbreviations are used in this manuscript:

FEM	Finite element method
GW	Guided wave
PWAT	Piezoelectric-wafer active transducer
DI	Damage index/indicator
SAHA	Semi-analytical hybrid approach
SBC	Spring boundary condition
